# Post-marketing surveillance of radium-223 chloride in Japanese patients with castration-resistant prostate cancer with bone metastasis—final analysis of 3-year extended follow-up focusing on bone fractures

**DOI:** 10.1007/s10147-025-02846-7

**Published:** 2025-08-08

**Authors:** Naoya Masumori, Makoto Hosono, Shunji Takahashi, Yoshiyuki Kakehi, Hirotsugu Uemura, Toshiyuki Sunaya, Kako Shimotsumagari, Yasuhiro Matsuba, Masatoshi Adachi, Haruka Kakiuchi, Seigo Kinuya

**Affiliations:** 1 Department of Urology, Sapporo Medical University School of Medicine, S1, W16, Chuo-ku, Sapporo, 060-8543 Japan; 2 Department of Radiology, Faculty of Medicine, Kindai University, 377-2 Ohno-Higashi, Osaka-Sayama, Osaka 589-8511 Japan; 3 Department of Medical Oncology, Cancer Institute Hospital of Japanese Foundation for Cancer Research, 3-8-31 Ariake, Koto-ku, Tokyo, Japan; 4 Department of Urology, Faculty of Medicine, Kagawa University, 1750-1, Ikenobe, Miki-cho, Kita-Gun, Kagawa 761-0793 Japan; 5 Department of Urology, Kindai University School of Medicine, 377-2 Ohno-Higashi, Osaka-Sayama, Osaka 589-8511 Japan; 6 Clinical Statistics and Analytics Japan, Research Development Japan, Bayer Yakuhin, Ltd., 2-4-9 Umeda, Kita-ku, Osaka, 530-0001 Japan; 7 PMS, Integrated Evidence Generation. Medical Affairs and Pharmacovigilance, Bayer Yakuhin, Ltd., 2-4-9 Umeda, Kita-ku, Osaka, 530-0001 Japan; 8 Pharmacovigilance Monitoring and Governance, Medical Affairs and Pharmacovigilance, Bayer Yakuhin, Ltd., 2-4-9 Umeda, Kita-ku, Osaka, 530-0001 Japan; 9 Medical Affairs Oncology, Medical Affairs and Pharmacovigilance, Bayer Yakuhin, Ltd., 2-4-9 Umeda, Kita-Ku, Osaka, 530-0001 Japan; 10 Department of Nuclear Medicine, Faculty of Medicine, Institute of Medical, Pharmaceutical and Health Sciences, Kanazawa University, 13-1 Takara-Machi, Kanazawa, 920-8641 Japan

**Keywords:** Bone metastases, Castration-resistant prostate cancer, Japanese patients, Post-marketing surveillance, Radium-223, Real-world data

## Abstract

**Background:**

A post-marketing surveillance (PMS) study was conducted in Japan to assess real-world outcomes with radium-223 treatment in men with metastatic castration-resistant prostate cancer (mCRPC). Results from the treatment period showed that radium-223 was generally well tolerated. Follow-up was subsequently extended to 3 years to collect data on fracture events. Results of the extended follow-up are now reported.

**Methods:**

This prospective, non-interventional, multicenter, single-cohort PMS study enrolled men with CRPC and bone metastases treated with radium-223 under clinical practice. Extended follow-up lasted until 3 years after the first administration of radium-223. Data on clinical fractures and survival were collected.

**Results:**

A total of 334 patients were enrolled, with a median follow-up of 15.3 months (range 1–50). The overall incidence proportion of fractures reported as adverse events was 7.76% (95% confidence interval [CI] 5.09–11.25%), with a fracture incidence rate of 5.22 patients [with fracture]/100 person-years (PY). Patients who received bone-modifying agents (BMAs) had a numerically lower incidence of fractures (5.85%; 3.46/100PY vs 9.93%; 7.92/100PY). Median overall survival was 26.32 months (95% CI 21.65-not reached).

**Conclusion:**

Compared with existing reference data, there was no obvious increase in the incidence of clinical fractures in Japanese patients with mCRPC who were treated with radium-223 under clinical practice. As is already well known for androgen deprivation, BMAs may also be useful in reducing bone fracture after radium-223.

**Supplementary Information:**

The online version contains supplementary material available at 10.1007/s10147-025-02846-7.

## Introduction

Bone is a common site of metastasis in men with advanced prostate cancer [1, 2]. Skeletal-related events (SREs) caused by bone metastases reduce patients’ quality of life and are associated with increased mortality [3–6]. Therefore, treatment goals in men with castration-resistant prostate cancer (CRPC) with bone metastases include the prevention of SREs and prolongation of survival.

Radium-223 dichloride is the only alpha-emitting radiopharmaceutical approved for the treatment of bone metastatic CRPC. It is incorporated into bone regions with an enhanced osteoblastic response, such as bone metastases [7, 8], and emits short-ranged alpha particles to exert a localized antitumor effect with limited toxicity to surrounding benign tissue such as bone marrow [7].

In the phase 3 ALSYMPCA trial in patients with symptomatic CRPC with bone metastasis, radium-223 significantly prolonged overall survival (OS) compared with placebo when added to best standard of care (median 14.9 vs 11.3 months, *p* < 0.001) and was generally well tolerated [9]. It also prolonged time to first symptomatic skeletal event (SSE; 15.6 vs 9.8 months, *p* < 0.001). Among the individual components of SSE, symptomatic pathological fracture tended towards a reduction (HR 0.62) with radium-223, although not achieving statistical significance (*p* = 0.10). An increased incidence of fracture was not reported in the ALSYMPCA trial, or in the Japanese phase 2 study [9–11] and the real-world observational REASSURE study [12, 13].

Unexpectedly, the phase 3 ERA-223 trial in patients with asymptomatic/mildly symptomatic CRPC showed an increased incidence proportion of bone fractures when radium-223 was combined with abiraterone acetate plus prednisone/prednisolone (AAP) compared with AAP alone (29% vs 11%) [14], while no significant between-group differences were shown with respect to median SSE-free survival or OS. Most fractures occurred outside of sites with bone metastasis, and the use of bone-modifying agents (BMAs) at baseline was associated with a lower incidence of fracture. Based on the results of the ERA-223 trial, the combination of AAP and radium-223 is not recommended.

A post-marketing surveillance (PMS) study of radium-223 in Japan started after it was launched in the country in 2016, with the primary objective of evaluating safety. Results for the treatment period have been reported previously, confirming that radium-223 was generally well tolerated [15, 16]. Five of the 296 participants experienced fractures, but none were considered related to radium-223 [16]. Because of the increased incidence of fracture seen when radium-223 was combined with AAP in the ERA-223 study, the follow-up period in the Japanese PMS study was extended to 3 years after the start of treatment, to collect data on fracture events. This also enabled survival data to be captured. Results of the final analysis, after extended follow-up, are reported here.

## Patients and methods

### Study design

A prospective, non-interventional, multicenter, single-cohort PMS study was performed in Japan (ClinicalTrials.gov NCT02803437). The study enrolled patients with CRPC and bone metastasis who were treated with radium-223 under clinical practice. Radium-223 was administered at a dose of 55 kBq/kg every 4 weeks for up to six cycles.

The target sample size was 300 patients, with enrollment taking place over an 18-month period. The observation period for the primary study was 6 months, from the first administration of radium-223 until 30 days after the last administration. The extended follow-up period started at the end of the primary study period and lasted until 3 years after the first administration of radium-223.

The study was conducted in accordance with Japanese regulations on Good Post-marketing Study Practice. Written informed consent from patients was not required, in accordance with Japanese regulations.

### Data collection

During the primary study period, data on treatment-emergent adverse events, and alkaline phosphatase (ALP) and prostate-specific antigen (PSA) levels were collected; results have been reported previously [15, 16].

During the extended follow-up period, data on clinical fractures were collected: presence/absence of fracture, type (pathological or non-pathological, consistency with metastatic lesion was not mandated), site, date of occurrence, CTCAE grade, outcome, seriousness, radium-223 causality, potential associated factors (e.g. osteoporosis), and treatment of fracture. Of note, adverse events aside from fracture were not mandated to be reported during this period. In addition, patient status (alive/dead), and information on treatments given during follow-up after completion (6 injections) or discontinuation of radium-223 treatment, were collected.

### Statistical analysis

Fracture events throughout the observation period were summarized descriptively, including number of events or patients among the overall study population (safety analysis set, comprising patients who received at least one injection of radium-223) and for pre-defined subgroups. Fracture incidence rate per person-year (PY) was calculated by dividing the number of patients with fractures by the total observation period (from first radium-223 injection to the end of the observation period or first fracture, if any) and was compared by exact Poisson test (two-sided).

The time course of fracture occurrence was represented by cumulative incidence plots, with fracture as the event and death as a competitive risk. Survival was estimated by Kaplan–Meier curves in the overall population and in subgroups. The incidence of fractures after treatment with radium-223 was analyzed according to known risk factors (based on the Japanese osteoporosis guidelines [17]).

OS was defined from the start of radium-223 treatment to the date of reported death and censored at the end of observation. OS according to percent change in ALP, PSA, and their combination at 12 weeks was analyzed using landmark analysis for patients who were alive at 12 weeks.

Prognostic factors for OS were exploratorily sought using multivariate analysis with Cox proportional hazard model, which are detailed in Supplementary Methods.

## Results

Between June 2016 and November 2017, 334 patients were enrolled from 128 sites. The safety analysis set comprised 322 patients who received at least one dose of radium-223. Median follow-up was 15.3 months (range 1–50).

Most patients had an ECOG performance score of 0, most were in category 0 of the WHO analgesic ladder, and almost half had a Gleason score of 9–10 (Table 1). Extent of disease categories 1, 2, and 3 were present in approximately one-third of patients each. Treatment line for radium-223 was evenly distributed between first, second, and third or later lines. Almost 70% of patients completed six doses of radium-223.

**Table 1:** Patient characteristics and prior/concomitant treatment

Parameter	Category	Safety analysis dataset (*n* = 322)
Duration of follow-up (months), median (range)		15.3 (1–50)
Age (years), median (range)		74.0 (48–93)
Body weight (kg), median (range)		63.0 (36.0–96.0)
ECOG PS (%)	0/1/≥ 2	70.5/25.2/4.4
WHO analgesic ladder (%)	0/1/≥ 2	67.7/22.7/9.6
Gleason score (%)	≤ 7/8/9–10	20.2/21.4/47.8
Extent of disease (%)	1/2/3/4^a^	28.9/29.5/31.7/2.8
PSA (ng/mL), median (range)		20.7 (0.0–5800)
ALP (U/L), median (range)		272.5 (76–4761)
Previous curative therapy (%)	Yes	31.7
Treatment line of radium-223 (%)	1 st/2nd/≥ 3rd	31.7/30.8/37.6
Number of radium-223 doses (%)	6/1–5	69.6/30.4

Prior/concomitant treatment	Prior	Concomitant^b^
First generation antiandrogens (%)	94.4	9.9
Androgen-receptor signaling inhibitors (%)	71.4	33.9
Enzalutamide	56.8	18.9
Abiraterone	46.9	17.1
Chemotherapy (%)	42.2	1.2
Docetaxel	40.4	0.6
Cabazitaxel	11.5	0.3
Bone-modifying agents (%)	65.5	44.4
Use after start of radium- 223, before onset of fracture^c^		53.1
Analgesics (NSAID) (%)	28.9	29.2
Analgesics (opioids) (%)	16.5	20.5
External beam radiation therapy (%)	18.6	3.1

^a^Extent of disease (EOD): < 6 bone metastases = EOD1; ≥ 6 to ≤ 20 = EOD2; > 20 = EOD3; Superscan = EOD4

^b^Duration of administration overlapping with duration of radium-223 treatment (including continuation from prior treatment)

^c^Received bone-modifying agents at any point between the start of radium-223 treatment and the onset of fracture (if any) or end of observation

*ALP* alkaline phosphatase, *ECOG PS* Eastern Cooperative Oncology Group performance status, *NSAID* non-steroidal anti-inflammatory drug, *PSA* prostate specific-antigen

The most common prior treatments other than androgen-depleting therapy (ADT) were first-generation antiandrogens (94.4%), androgen receptor-signaling inhibitors (ARSIs, including enzalutamide and abiraterone acetate: 71.4%/56.8%/46.9%), chemotherapy (42.2%), and BMAs (65.5%). The most common concomitant treatments (duration of administration overlapping with duration of radium-223 treatment) were ARSIs/enzalutamide/abiraterone acetate (33.9%/18.9%/17.1%), and BMAs (44.4%). Overall, 53.1% of patients received BMAs at some point between the start of radium-223 treatment and the onset of fracture (if any) or end of observation.

### Fracture

**Fig. 1:**
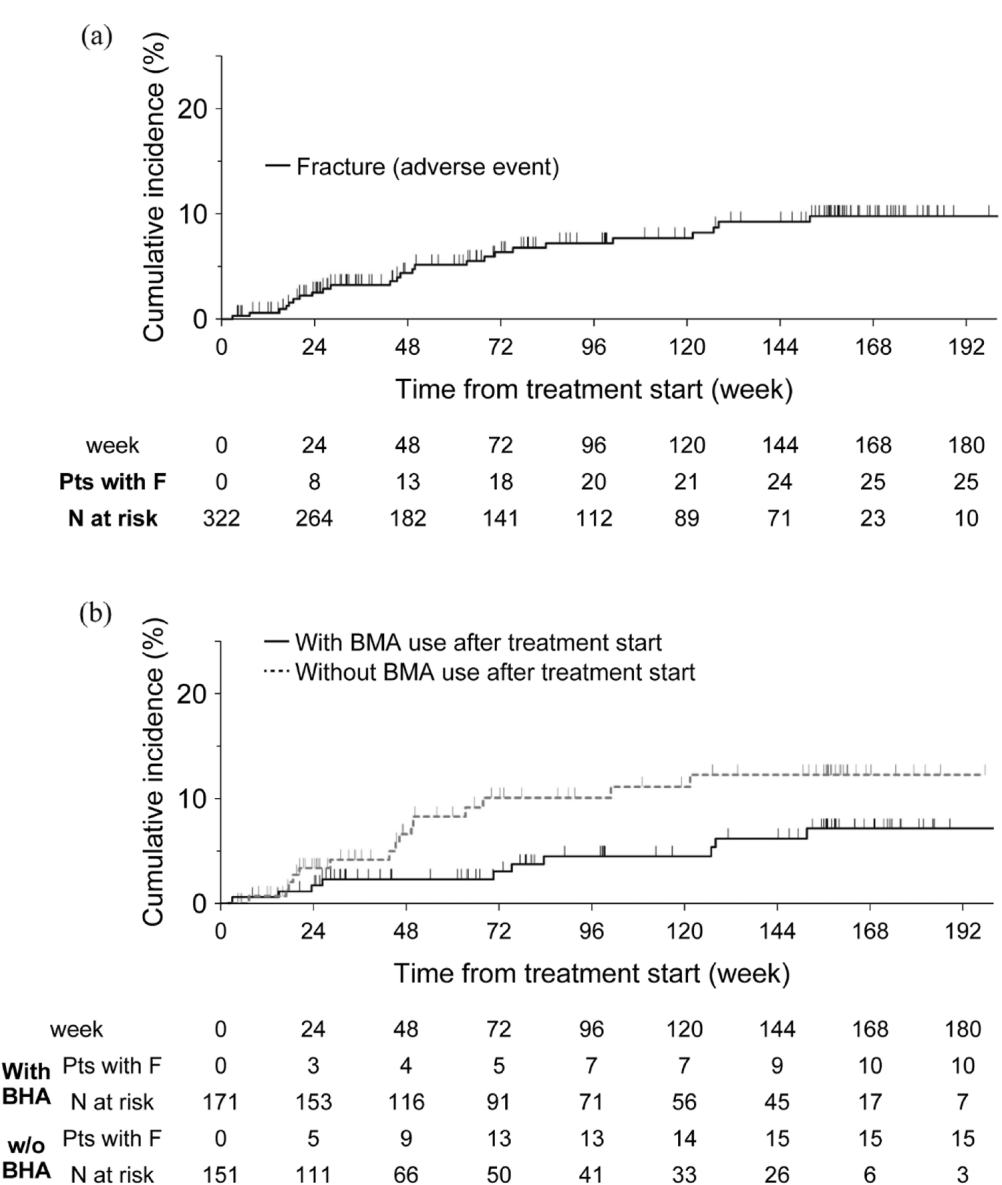
Time course of fracture occurrence **a** Total study population **b** According to use/non-use of bone-modifying agents (BMA)

Four cases with fracture (including 1 case with 2 events) were reported as adverse reactions (i.e. causality could not be ruled out by the investigator and/or the company; 1.24%, 95% confidence interval [CI] 0.34–3.15%). The adverse reaction fracture incidence rate was 0.80 patients per 100PY (Table 2). As adverse events (i.e. irrespective of causality), fractures were reported in 25 patients (7.76%, 5.09–11.25%, 31 fractures), in more than half of whom (15/25) the event was grade 1 or 2. The adverse event fracture incidence rate was 5.22 patients per 100PY. Regarding the site of fracture, vertebrae was the most common (Table 2). Of the 25 patients, 3 fractures (one G3, two G2) reported in skull bone were suspected to be osteonecrosis of the jaw (ONJ). In addition, 3 more ONJ events were reported as adverse events (not related to radium-223) in 3 other patients during the treatment period through which all adverse events were reported. All these 6 patients had received BMA. Pathological fractures were numerically more common (15/322 patients; 4.7%, 95% CI 2.63–7.57%) than non-pathological fractures (9/322 patients; 2.8%, 95% CI 1.29–5.24%). However, judgment on fracture type was at the discretion of the attending physician, and the presence of metastasis at the fracture site was not a requirement. Non-pathological fractures included femur fractures in 3 patients; one at the femoral neck and one at the trochanter were associated with falls, and for one subtrochanteric fracture, prior use of zoledronic acid for 7 years was reported as a potential contributory factor. As for the time course, fractures occurred throughout the observation period and were not limited to any specific period, such as during and immediately after treatment (Fig. 1a).

**Table 2:** Incidence, grade, type, and site of fractures

Incidence and grade							
Fracture event	Number of patients (n/N)	Worst grade		SAE	Proportion of patients, % (95% CI)	Incidence (per 100 PY)	Number of events
		G1/2	G3				
Adverse reactions^a^	4/322	1	3	4	1.24 (0.34–3.15)	0.80	5
Adverse events^b^	25/322	15	9	10	7.76 (5.09–11.25)	5.22	31
Pathological fracture^c^	15/322	9	5	7	4.7 (2.63–7.57)	3.05	20
Non-pathological fracture^c^	9/322	5	4	3	2.8 (1.29–5.24)	1.82	10

Type and site of fracture (adverse events)		
		Number of patients
Site	Skull^d^	3/25
	Vertebrae	13/25
	Chest	2/25
	Pelvis	2/25
	Upper limb	2/25
	Lower limb	4/25
Type of fracture^c^	Pathological	15
	Non-pathological	9
	Femur fracture	3/9
	Jaw fracture	1/9
	Rib fracture	1/9
	Traumatic fracture	3/9
	Lumbar vertebra fracture	1/9
	Thoracic spine fracture	1/9

^a^Causal relationship could not be ruled out

^b^Irrespective of causality

^c^Type of fracture (pathological/non-pathological; information was lacking in 1 patient) was determined by the attending physician (assessment of consistency with bone metastasis was not required)

^d^All 3 skull bone fractures were described as related to osteonecrosis of the jaw, 2 were categorized as pathological and the other was categorized as a non-pathological fracture

*CI* confidence interval, *PY* person-year, *SAE* serious adverse event

**Fig. 2:**
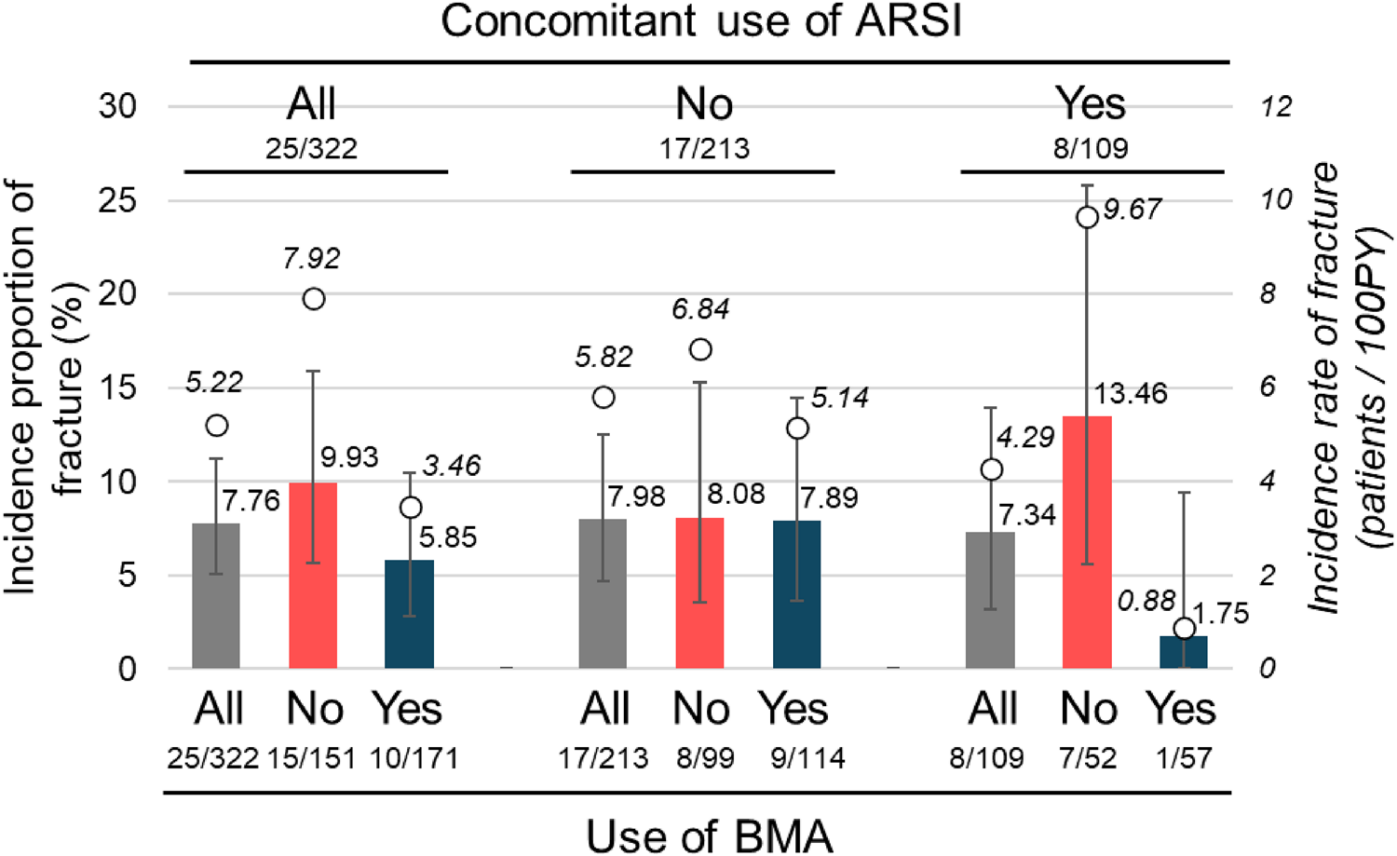
Fracture reported as adverse events according to the use of bone-modifying agents (BMA) and concomitant use of androgen-receptor signaling inhibitors (ARSI). Color bars indicate incidence proportion of fracture (%, left axis) and circles indicate incidence rate (patients/100PY, right axis). Error bars indicate 95% confidence intervals. Plain and italic numbers in the graph indicate the values of incidence proportion and incidence rate, respectively. Grouping by concomitant use of ARSI (top) and by use of BMA (bottom) are shown in the graph, respectively. Numbers below groups are of patients with fracture (numerator) and of total patients in the group (denominator)

The incidence of fracture events was evaluated further according to the use of BMAs and concomitant ARSIs (Fig. 2). Patients who received BMAs at least once from the start of radium-223 until the end of observation or the first fracture (if any), were considered as “use of BMAs”. BMA recipients had numerically lower fracture incidence proportion and rate (5.85 vs 9.93%; 3.46 vs 7.92 patients/100PY, *p* = 0.0423, exact Poisson test), as shown visually in Fig. 1b. However, limited events, potential confounding, and the post-hoc nature of this testing precluded statistical conclusions regarding BMA efficacy. A similar pattern was observed for patients receiving concomitant ARSIs (with vs without BMAs: 1.75 vs 13.46%, 0.88 vs 9.67 patients/100PY). When individual ARSIs were considered, the incidence proportions of fracture among the overall, with BMA use, and without BMA use populations were 12.7% (7/55), 4.2% (1/24), and 19.4% (6/31), respectively, for combination with abiraterone, and 1.6% (1/61), 0% (0/37), and 4.2% (1/24), respectively, for combination with enzalutamide. Patient background characteristics in each subgroup are described in Supplementary Table 1.

No obvious associations were apparent between known risk factors among baseline characteristics and increased fracture risk after radium-223 treatment, although the small event numbers did not allow for clear conclusions to be drawn (Supplementary Table 2). Elderly patients (> 75 years) tended to be at higher risk, but no relationships were seen for other factors (body mass index, extent of disease, prior history/concomitant use of external beam radiotherapy or corticosteroids). Patients who completed six doses of radium-223 appeared to have a higher incidence proportion of fracture than patients who received fewer doses (9.82% vs 3.09%), but these subgroups did not differ in terms of fracture incidence rate per PY (5.20 vs 5.40), suggesting the apparent effect may have been due to the difference in observation periods.

### Overall survival

**Fig. 3:**
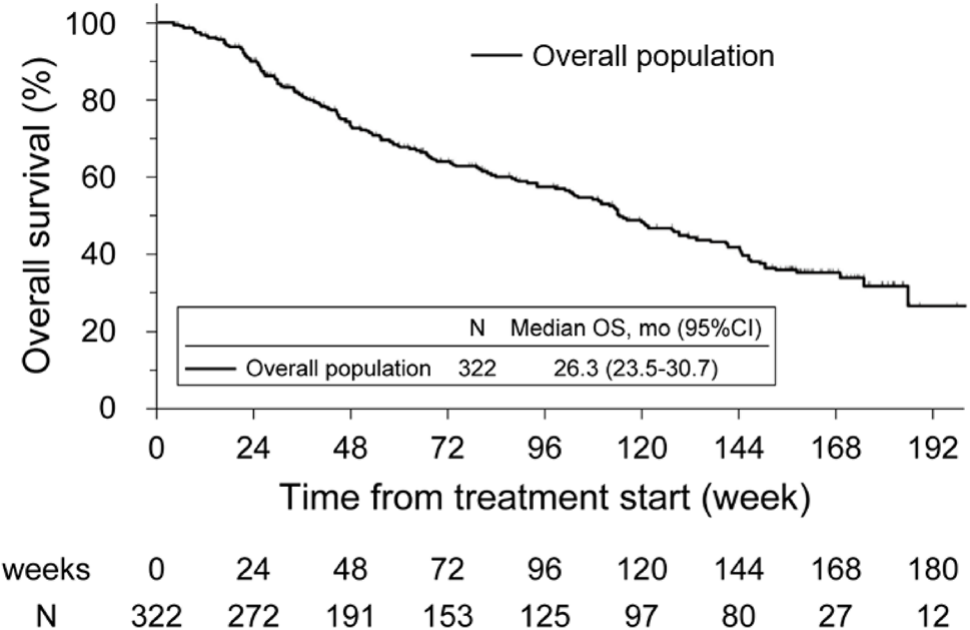
Overall survival (OS)

Median OS for the overall study population was 26.32 months (95% CI 21.65-not reached) (Fig. 3). Multivariate analysis identified six baseline characteristics as independent prognostic factors for OS, including number of bone metastases (extent of disease 3), prior history of taxanes (1 or 2 regimens), baseline PSA, baseline ALP, baseline red blood cell count, and baseline white blood cell count (Supplementary Table 3). These factors were largely consistent with known general prognostic factors in mCRPC. Kaplan–Meier curves according to independent prognostic factors are shown in Supplementary Fig. 1.

**Fig. 4:**
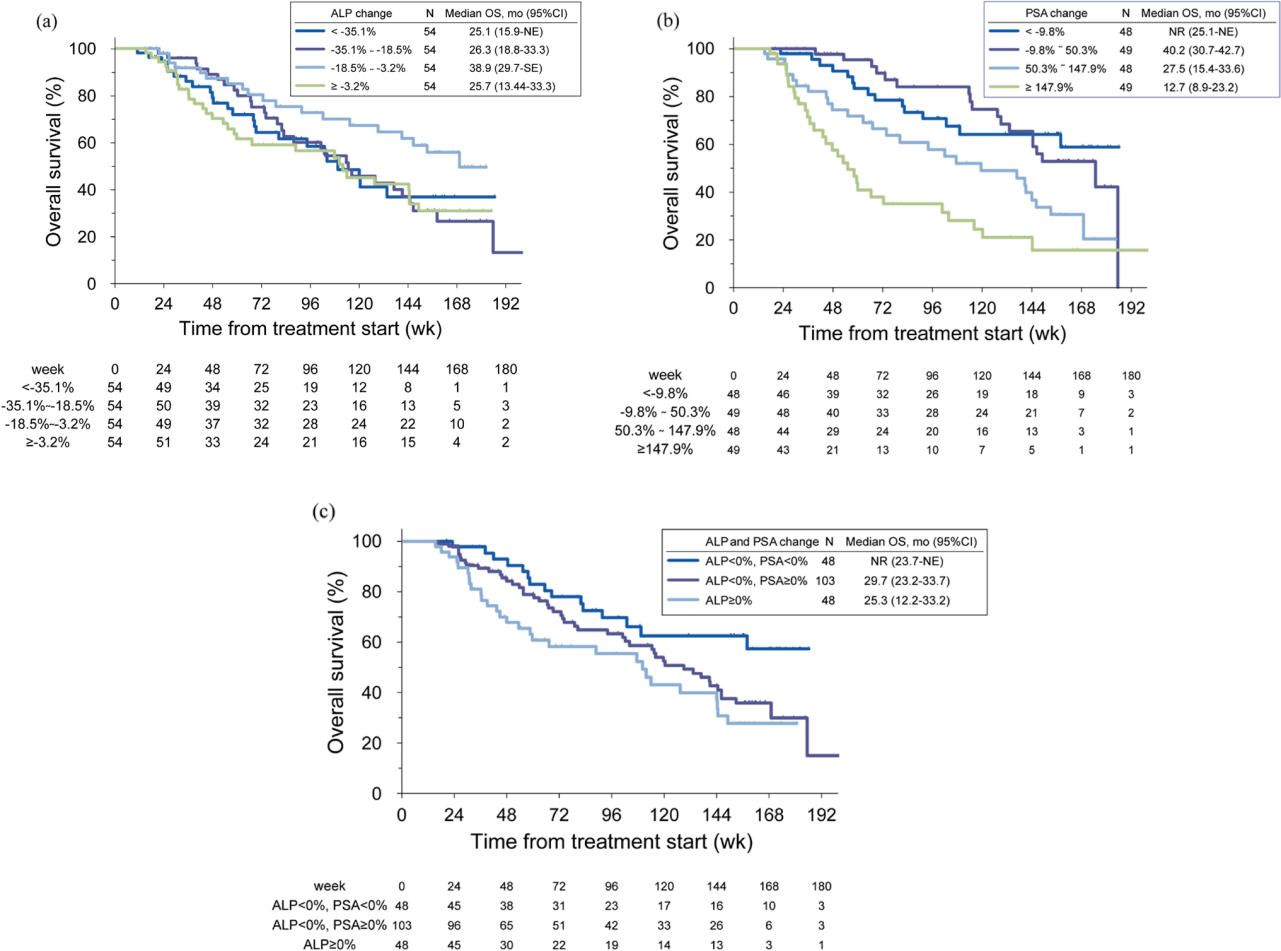
Overall survival (OS) according to change in **a** alkaline phosphatase (ALP), **b** prostate-specific antigen (PSA), and **c** ALP and PSA combined: landmark analysis in patients alive at week 12

OS according to percent change in laboratory parameters (ALP and PSA) at 12 weeks was analyzed by landmark analysis in patients who were alive at week 12. Kaplan–Meier curves by ALP, PSA, and their combination are shown in Fig. 4.

### Treatment after completion/discontinuation of radium-223

Two-thirds of patients received post-treatment with life-prolonging therapy (LPT, defined as docetaxel, cabazitaxel, abiraterone, and enzalutamide) after completion/discontinuation of radium-223 (Supplementary Table 4). The most frequent LPTs given first and second after radium-223 were an ARSI (170/205 patients) and taxane chemotherapies (59/92 patients), respectively. The most common pattern of BMA use was ‘prior-, concomitant-, and post-therapies’ (126/322 patients, 39.1%), followed by ‘never used’ (86/322 patients, 26.7%), and ‘only used as prior therapy’ (65/322 patients, 20%). Approximately half of all patients (156/322 patients, 48.5%) received BMAs after the completion/discontinuation of radium-223 treatment.

## Discussion

The current study reports data on fracture events and survival during up to 3 years of follow-up in a Japanese PMS study of radium-223. The overall incidence proportion of fracture adverse events was 7.76%, and median OS was 26.32 months.

Because the risk of fracture has been shown to increase as the duration of ADT increases [18], it would be expected to be higher in patients with CRPC who generally tend to be exposed to prior long-term ADT. Therefore, the absolute incidence proportion of fracture is proportionally affected by the duration of observation, making inter-study comparison difficult. In the current study, the occurrence of fracture after radium-223 treatment was not limited to a specific time-period and appeared relatively constant (Fig. 1), supporting the use of fracture incidence rate per PY to compare with/without radium-223 treatment. The fracture incidence rate after treatment with radium-223 was 7.92 patients per 100PY in the absence of BMA (Fig. 2). This value is within a similar range of fracture incidence rates reported in other observational studies of radium-223 such as REASSURE (4.34/100PY, [13]) and FLATIRON (11/100PY, [19]). For a comparison of fracture incidence rate in the absence of radium-223 use, reference data are available from a large Swedish cohort study (FRAILCO; *n* = 179,744), in which the fracture incidence rate of patients with prostate cancer not receiving radium-223 (or only a few, if any, due to the 2008–2014 study registration period), all receiving ADT and 4.1% receiving BMA, was reported to be 74/1000PY (n = 6954) [20]. This is very close to the fracture incidence rate in the current PMS study (7.92/100PY), suggesting that the use of radium-223 did not lead to an obvious increase in fracture incidence rate within a period of up to 3 years after treatment start. This is consistent with the phase 3 ALSYMPCA trial [9] and with large observational studies of radium-223, in which an obvious increase in fracture incidence was not reported [13, 19, 21]. Recently, a meta-analysis of randomized trials in men with prostate cancer reported that the relative risk (1.59; 95% CI, 1.35–1.89; *p* < 0.001) of all-grade fracture increased with androgen receptor inhibitors (ARIs) versus the control group (patients could have received placebo, bicalutamide, or abiraterone) [22]. Considering this evidence, the risk of fracture after radium-223 may not require additional, specific attention compared with other CRPC drugs.

The effects of ARSI on the incidence of fracture in the current study (Fig. 2) were largely similar to those in the phase 3 ERA-223 and PEACEIII studies [14, 23]. In the ERA-223 study, the risk of fracture was increased in the AAP plus radium-223 arm versus the AAP arm and was lower in patients receiving concomitant BMAs in both arms [14]. Also, interim safety analysis of PEACEIII showed that the incidence proportion of fracture was numerically higher in the enzalutamide plus radium-223 arm versus the enzalutamide arm in the absence of BMA use (45.9 vs 21.9% at 18 months), but it was well controlled in both arms in the presence of BMAs (4.3 vs 2.6% at 18 months) [23]. The current study observed numerically lower fracture incidence rates with BMA (3.46 vs 7.92/100PY) and, in the absence of BMAs, numerically higher fracture incidence rates (9.67 vs 6.84/100PY) with ARSI, consistent with findings from ERA-223 and PEACEIII [14, 23]. These results, while statistically equivocal, tend towards consistency with other results supporting the use of BMAs with radium-223, as recommended by several guidelines [24–26].

When comparing individual ARSIs, the effects of concomitantly administered AAP and enzalutamide were different in the current study. It is intriguing to associate this with differences in preclinical results showing that the combination of radium-223 with AAP increased bone resorption indicated by TRACP 5b [27], but not the combination of radium-223 with enzalutamide [28]. However, because the use of ARSIs was not controlled or stratified in this PMS, it leaves room for confounding, and does not allow conclusive discussion. On the other hand, regarding corticosteroid use, a known risk factor for fracture that is also included in AAP regimens, no obvious increase in fracture was observed after radium-223 according to corticosteroid prior history or concomitant use (Supplementary Table 1).

With respect to effectiveness, median OS in the overall population (26.3 months) was numerically longer than that reported for clinical studies such as ALSYMPCA (14.9 months), the Japanese phase 2 study (19.3 months), the international expanded access program (EAP; 16 months) and the US EAP (17 months) [9, 11, 29, 30]. This may be partially attributable to the differences in patient populations or availability of other treatments. For example, most patients in the current study were treated with radium-223 relatively early in the disease course (31.7% and 30.8% were receiving first- or second-line mCRPC treatment, respectively) whereas, in the US EAP, 99% of patients had received prior therapies [30]. Risk factors for poor OS identified in this PMS included EOD 3, prior history of taxanes, and high (PSA, ALP, and WBC) and low (RBC) laboratory values at baseline. Recently, Sasaki et al. [31], reported inability to complete 6-cycles of radium-223, as well as PS > 0, PSA > 10 ng/mL, and EOD 3–4 were negatively prognostic to OS, which is partially similar to our results.

Prediction of survival outcome by biomarker response was also explored. Prognosis appeared to be better differentiated by PSA change, differing from post-hoc analysis results from the ALSYMPCA population, in which any decrease in ALP correlated with OS, while PSA change did not [32]. This could be partly due to potential confounding by the concomitant use of ARSIs in one-third of patients in the PMS population. When changes in ALP and PSA were combined, patients with ALP change > 0% had the worst OS; those with ALP < 0% were split according to PSA change (increase vs decrease). It should be noted that the population included in the landmark analysis was limited to those surviving at 12 weeks, leaving room for selection bias.

The main limitation of the study is the single-cohort design. However, the analysis reflects patients treated in a real-world clinical setting, rather than the carefully selected population that would be enrolled in a controlled clinical trial. Therefore, the results are applicable to patients treated in clinical practice in Japan. In addition, the inhibitory effect of BMA was not proven by multivariate analysis, leaving the possibility of confounding by other risk factors. Nevertheless, similar results reported elsewhere [14, 23] are in line with our results.

In conclusion, this extended 3-year PMS follow-up examined the incidence of clinical fractures and prognosis in Japanese patients with mCRPC who received radium-223 in clinical practice. Compared with existing reference data, there was no obvious increase in the incidence of clinical fracture after treatment with radium-223. The incidence of fractures after radium-223 treatment may be reduced using BMAs, in agreement with guideline recommendations.

## Supplementary Information

Below is the link to the electronic supplementary material.

Supplementary file1 (DOCX 418 KB)
