# Supplementary material for: Post-marketing surveillance of radium-223 chloride in Japanese patients with castration-resistant prostate cancer with bone metastasis—final analysis of 3-year extended follow-up focusing on bone fractures
Source: Int J Clin Oncol. 2025 Aug 8;30(10):2118–27. doi: 10.1007/s10147-025-02846-7 (PMC12474616; doi:10.1007/s10147-025-02846-7)

**Electronic Supplementary Material**

**International Journal of Clinical Oncology**

**Post-marketing surveillance of radium-223 chloride in Japanese patients with castration-resistant prostate cancer with bone metastasis – final analysis of 3-year extended follow-up focusing on bone fractures**

**Authors:** Naoya Masumori, Makoto Hosono, Shunji Takahashi, Yoshiyuki Kakehi, Hirotsugu Uemura, Toshiyuki Sunaya, Kako Shimotsumagari, Yasuhiro Matsuba, Masatoshi Adachi, Haruka Kakiuchi, Seigo Kinuya

**Corresponding author:** Naoya Masumori, Department of Urology, Sapporo Medical University School of Medicine, S1, W16, Chuo-ku, Sapporo, 060-8543, Japan. E-mail: [masumori@sapmed.ac.jp](mailto:masumori@sapmed.ac.jp)

**Supplementary Methods**

**Multivariate analysis of prognostic factors for OS**

In an exploratory analysis, prognostic factors for OS were sought using multivariate analysis with Cox proportional hazard model. Baseline clinical factors used for multivariate analysis were selected based on clinical relevance, and those with the following features were excluded: ≥10% missing values, contained levels with <5% of the population, hierarchical structure between levels, or multicollinearity. Data availability was targeted at 70% (actual 71.2%). Factors fulfilling significance (<5%, two-sided) were then sought with the forward-backward stepwise method.

**Supplementary Table 1. Patient background factors categorized by the absence or presence of bone-modifying agents**


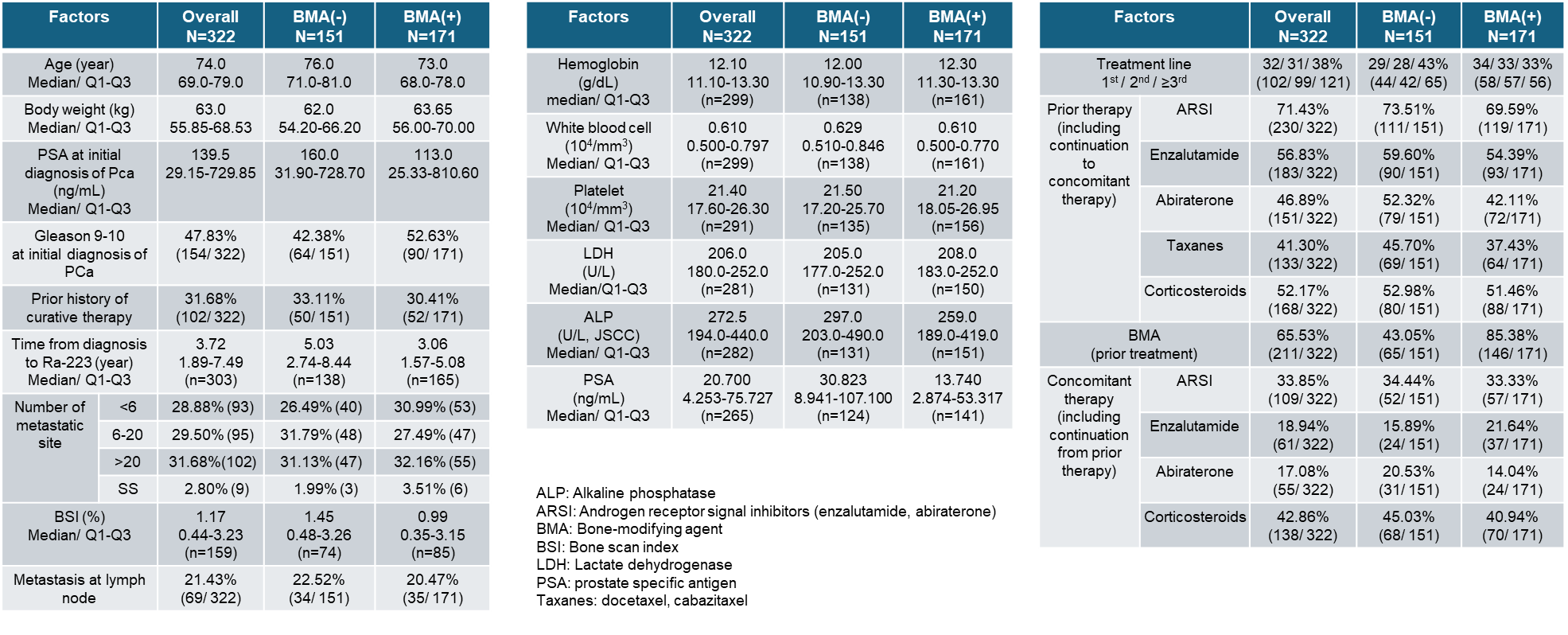


**Supplementary Table 2 Fractures (adverse events) according to background risk factors**

| **Background factor** |  | **Patients with fracture (n/N)** | **Proportion of patients with fracture, % (95% CI)** | **Incidence rate**  **(patients/**  **100PY)** | **Number of fracture events** |
| --- | --- | --- | --- | --- | --- |
| Overall |  | 25 / 322 | 7.76% (5.09- 11.25) | 5.22 | 31 |
| Age | <75 years old | 10 / 162 | 3.89% (3.00- 11.06) | 3.89 | 12 |
|  | ≥75 years old | 15 / 160 | 6.77% (5.34-14.99) | 6.77 | 19 |
| BMI^a^ | <18.5 | 0 / 23 | 0.00% (0.00-14.82) | 0.00 | 0 |
|  | ≥18.5 to <25 | 17 / 197 | 8.63% (5.11-13.46) | 6.09 | 19 |
|  | ≥25 | 7 / 79 | 8.86% (3.64-17.41) | 4.73 | 11 |
| Extent of disease (number of bone metastatic lesions) | <6 | 7 / 93 | 7.53% (3.08-14.90) | 3.91 | 10 |
|  | 6-20 | 10 / 95 | 10.53% (5.16-18.51) | 6.78 | 11 |
|  | >20 | 6 / 102 | 5.88% (2.19-12.36) | 5.55 | 8 |
|  | Super scan | 1 / 9 | 11.11% (0.28-48.25) | 11.20 | 1 |
| EBRT | Prior history Yes    No | 4 / 60 | 6.67% (1.85-16.20) | 6.12 | 4 |
|  |  | 21 / 262 | 8.02% (5.03- 11.99) | 5.08 | 27 |
|  | Concomitant use Yes    No | 0 / 10 | 0.00% (0.00-30.85) | 0.00 | 0 |
|  |  | 25 / 312 | 8.01% (5.25-11.60) | 5.35 | 31 |
| Corticosteroids | Prior history Yes    No | 12 / 168 | 7.14% (3.75-12.14) | 5.41 | 14 |
|  |  | 13 / 154 | 8.44% (4.57-14.00) | 5.06 | 17 |
|  | Concomitant use Yes    No | 12 / 138 | 8.70% (4.57-14.70) | 6.55 | 14 |
|  |  | 13 / 184 | 7.07% (3.82-11.78) | 4.40 | 17 |
| Number of radium-223 doses | 6 | 22 / 224 | 9.82% (6.26-14.49) | 5.20 | 28 |
|  | 1-5 | 3 / 98 | 3.09% (0.64-8.69) | 5.40 | 3 |

^a^ One patient with unknown BMI.

*CI* confidence interval; *BMI* body mass index; *EBRT*, external beam radiotherapy (to relieve skeletal symptoms); *PY* person-years.

**Supplementary Table 3 Factors associated with overall survival (Cox proportional hazard model)**

| **Explanatory variable ^a^** | **Hazard ratio** | **95% CI**  **lower limit** | **95% CI upper limit** | **P-value** |
| --- | --- | --- | --- | --- |
| Number of bone metastases, >20 vs <6 | 1.859 | 1.246 | 2.774 | 0.0024 |
| Prior taxane treatment, 1 vs none | 1.554 | 1.009 | 2.394 | 0.0454 |
| Prior taxane treatment, 2 vs none | 3.276 | 1.667 | 6.439 | 0.0006 |
| Log_10_ PSA (ng/mL) | 1.493 | 1.170 | 1.905 | 0.0013 |
| Log_10_ ALP (U/L) | 5.527 | 2.876 | 10.624 | <0.0001 |
| Red blood cell count | 0.992 | 0.987 | 0.996 | <0.0001 |
| White blood cell count | 2.110 | 1.121 | 3.974 | 0.0207 |

*ALP* alkaline phosphatase; *CI* confidence interval; *PSA* prostate specific-antigen.

a: the analysis started with the following baseline factors: number of bone metastatic lesions, WHO analgesic ladder, prior history of taxanes, number of life-prolonging therapies before radium-223, PSA, ALP, lactate dehydrogenase, red blood cell count, white blood cell count, and ECOG performance score.

**Supplementary Table 4 Life-prolonging therapies administered after completion/discontinuation of radium-223 treatment**

|  | **Sum of all LPTs after radium-223** | | **1st LPT after radium-223** | | **2nd LPT after radium-223** | | **3rd LPT after radium-223** | |
| --- | --- | --- | --- | --- | --- | --- | --- | --- |
|  | **(n)** | **mDOT, wk (range)** | **(n)** | **mDOT, wk (range)** | **(n)** | **mDOT, wk (range)** | **(n)** | **mDOT, wk (range)** |
| LPT | 205/322 | 47.71 (18.14-102.43) | 205 | 18.86 (7.29-54.14) | 92 | 17.93 (9.21-39.93) | 34 | 14.64 (10.86-29.14) |
| ARSI | 178 | 27.93 (10.00-70.00) | 170 | 21.57 (8.00-61.57) | 33 | 19.00 (15.14-48.14) | 9 | 11.00 (4.29-14.00) |
| Enzalutamide | 108 | 18.43 (8.50-54.43) | 83 | 24.00 (8.14-59.86) | 18 | 18.43 (15.14-72.14) | 3 | 12.14 (11.00-14.00) |
| Abiraterone acetate | 109 | 19.00 (8.00-53.43) | 87 | 20.29 (6.57-74.86) | 15 | 20.14 (10.00-44.57) | 6 | 5.14 (3.14-17.43) |
| Taxane chemotherapy | 96 | 18.21 (7.64-50.07) | 35 | 12.14 (5.86-31.14) | 59 | 17.00 (7.00-37.43) | 25 | 18.14 (12.14-37.14) |
| Docetaxel | 65 | 17.00 (6.14-28.86) | 24 | 10.79 (4.64-27.07) | 34 | 17.71 (8.43-37.14) | 7 | 12.14 (8.14-20.29) |
| Cabazitaxel | 60 | 16.21 (8.64-37.64) | 11 | 13.14 (6.43-31.14) | 25 | 14.57 (7.00-39.14) | 18 | 21.57 (12.29-39.29) |

With respect to life-prolonging therapies including ARSI (abiraterone, enzalutamide) and taxane chemotherapy (docetaxel, cabazitaxel), the median duration of treatment when used as first to third life-prolonging therapy after radium-223, as well as the total length of use after radium-223 are shown.

*ARSI* androgen receptor-signaling inhibitor; *mDOT* median duration of treatment; *LPT* life-prolonging therapy; *Ra-223* radium-223; *wk* weeks.

**Supplementary Fig. 1 Median overall survival (OS) according to independent prognostic factors: number of bone metastases, prior history of taxanes, baseline prostate-specific antigen (PSA) level, baseline alkaline phosphatase (ALP) level, baseline red blood cell (RBC) count, and baseline white blood cell (WBC) count**
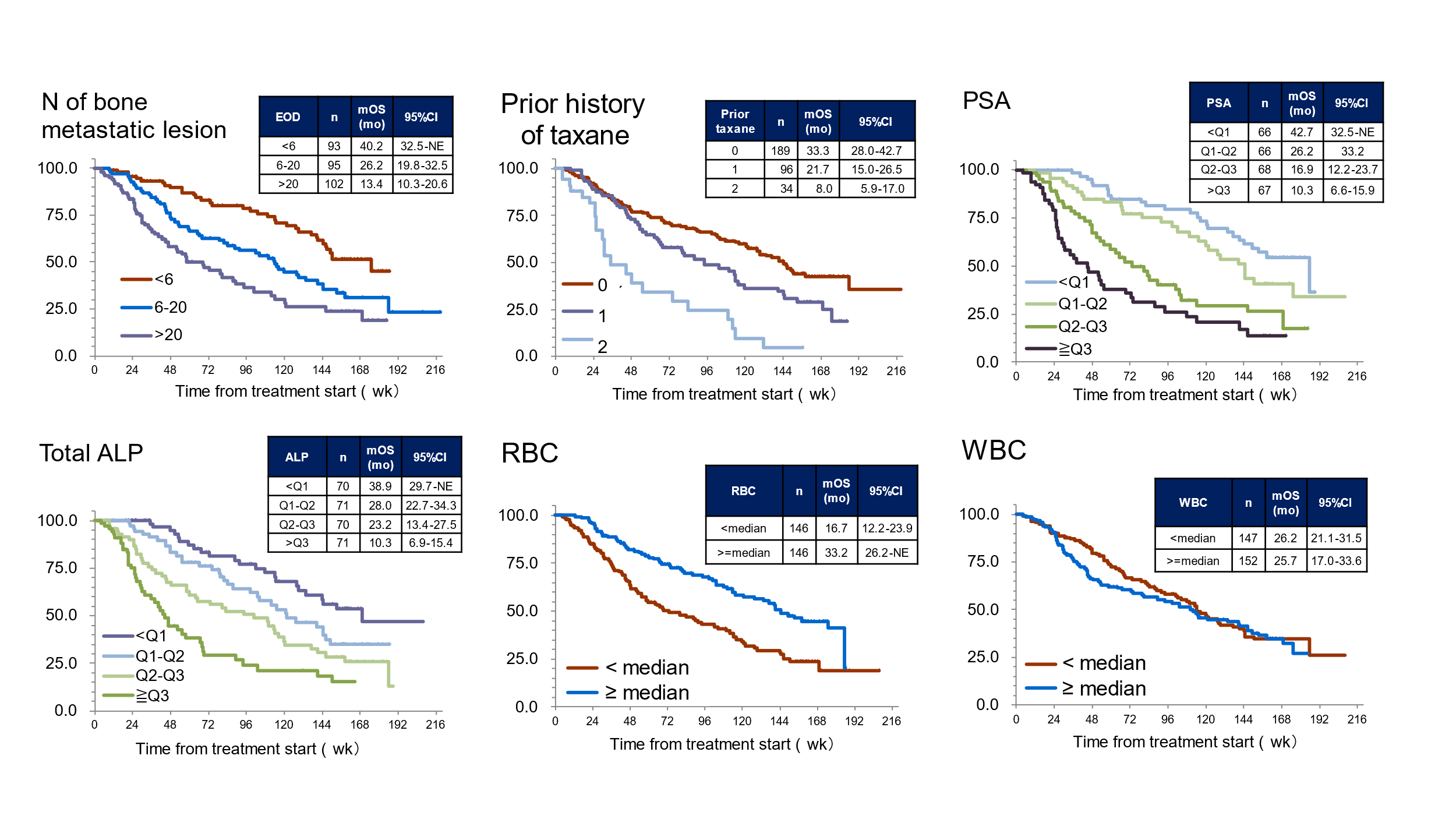

Supplement: Supplementary file 1 — Supplementary file1 (DOCX 418 KB) [file 10147_2025_2846_MOESM1_ESM.docx]
